# Treatment of Lp(a): Is It the Future or Are We Ready Today?

**DOI:** 10.1007/s11883-023-01141-y

**Published:** 2023-09-05

**Authors:** Alexandros D. Tselepis

**Affiliations:** Atherothrombosis Research Centre/Laboratory of Biochemistry, Department of Chemistry, University of Ioannina, 45110 Ioannina, Greece

**Keywords:** Antisense oligonucleotides, Apolipoprotein (a), Coronary artery disease, CRISPR/Cas9, Small interference RNAs, Atherosclerosis, Calcific aortic valve stenosis, Lipoprotein (a)

## Abstract

**Purpose of Review:**

The goal of this review is to present the pharmacodynamic effectiveness as well as the clinical efficacy and safety of investigational antisense oligonucleotides (ASOs) and small interference RNAs (siRNAs) drugs that specifically target lipoprotein(a) (Lp(a)). The review will discuss whether the existing lipid-lowering therapies are adequate to treat high Lp(a) levels or whether it is necessary to use the emerging new therapeutic approaches which are based on the current RNA technologies.

**Recent Findings:**

Lipoprotein(a) (Lp(a)) is a causal risk factor for atherosclerotic cardiovascular disease (ASCVD), independent of other conventional risk factors. High Lp(a) levels are also independently associated with an increased risk of aortic stenosis progression rate. Plasma Lp(a) levels are primarily genetically determined by variation in the LPA gene coding for apo(a). All secondary prevention trials have demonstrated that the existing hypolipidemic therapies are not adequate to reduce Lp(a) levels to such an extent that could lead to a substantial reduction of ASCVD risk. This has led to the development of new drugs that target the mRNA transcript of LPA and efficiently inhibit Lp(a) synthesis leading to potent Lp(a) reduction. These new drugs are the ASO pelacarsen and the siRNAs olpasiran and SLN360. Recent pharmacodynamic studies showed that all these drugs potently reduce Lp(a) up to 98%, in a dose-dependent manner. Ongoing clinical trials will determine the Lp(a)-lowering efficacy, tolerability, and safety of these drugs as well as their potential effectiveness in reducing the ASCVD risk attributed to high plasma Lp(a) levels.

**Summary:**

We are not ready today to significantly reduce plasma Lp(a). Emerging therapies potently decrease Lp(a) and ongoing clinical trials will determine their effectiveness in reducing ASCVD risk in subjects with high Lp(a) levels.

## Introduction

**Fig. 1:**
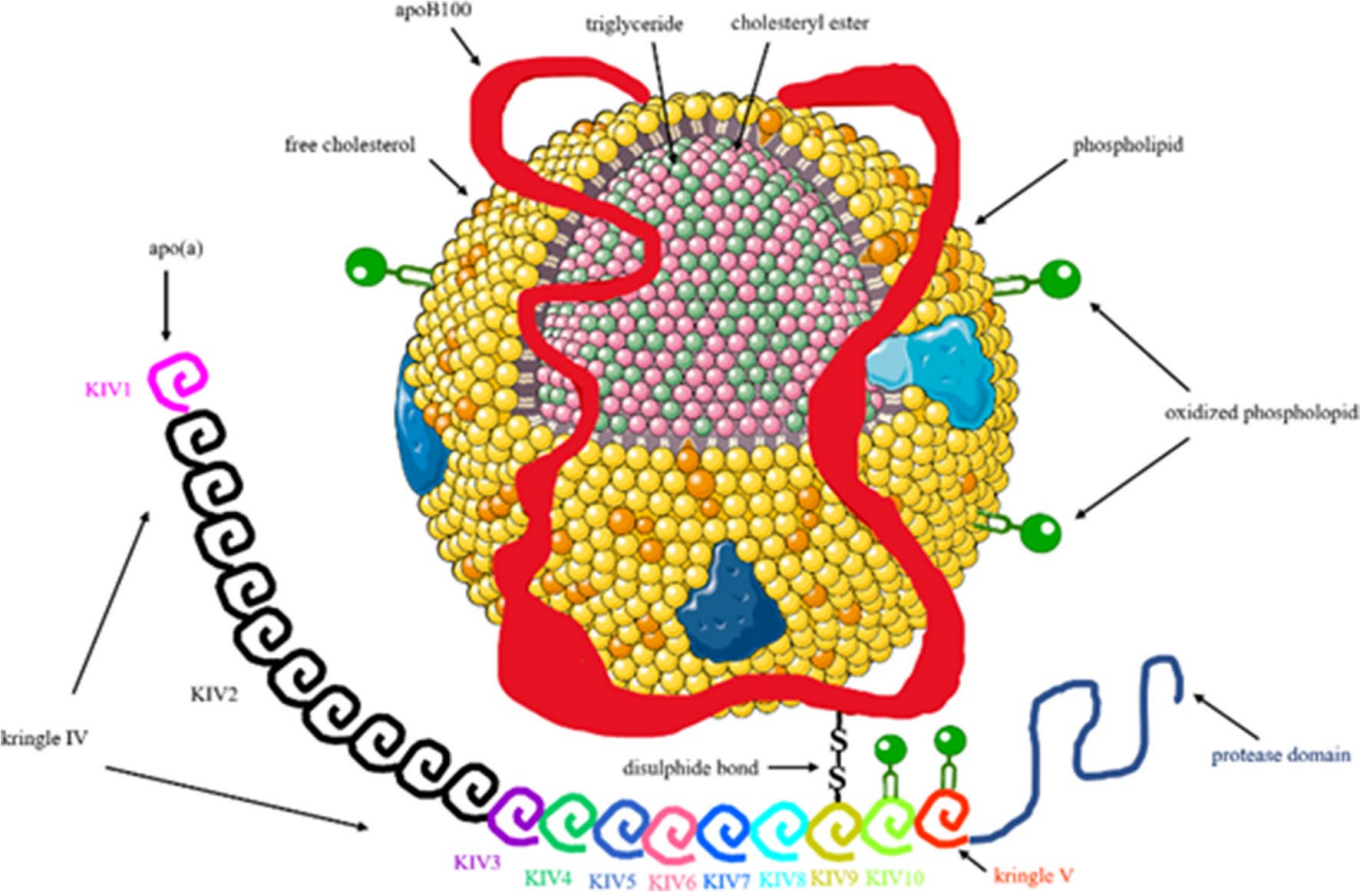
Structure of lipoprotein(a). Lipoprotein(a) consists of a low-density lipoprotein (LDL)-like particle, in which the apolipoprotein B-100 (ApoB-100) is linked by a single disulphide bridge to the glycoprotein apolipoprotein(a) [(Apo(a)]. Apo(a) contains 10 copies of kringle IV (KIV), 1 copy of KV, and a catalytically inactive protease domain. KIV contains 1 copy of the subtypes KIV1 and KIV3-10, but variable copies of KIV2, ranging from 1 to >40 on each allele. Up to 90% of all oxidized phospholipids found in human lipoproteins are carried on lipoprotein(a). (From *Eur J Clin Invest*. 2022;52:e13710, with permission from John Wiley and Sons) [4]

Lipoprotein(a) (Lp(a)) consists of a low-density lipoprotein (LDL)-like particle to which a large, highly glycosylated apolipoprotein(a) (apo(a)) is covalently bound to the apoB-100 moiety of LDL via a single disulfide bond [1–3] (Fig. 1). Apo(a) is highly homologous to the plasma protease zymogen, plasminogen, which contains five tri-loop structures stabilized by three disulfide bonds, named kringles (K), and a protease domain, thus it can be activated to plasmin. Apo(a) contains only KIV and KV and has a protease-like domain that is catalytically inactive, despite having an intact Ser-His-Asp catalytic triad [1–3]. Importantly, apo(a) contains 10 subtypes of KIV (KIV-1 to KIV-10); the KIV-2 subtype being present in variable numbers (5 to 50) of identically-repeated copies. Thus, apo(a) is highly polymorphic in length due to the number variation of the KIV-2 copies [1, 2]. Apo(a) is encoded by the LPA gene, which contains a 5.6-kb segment existing in multiple repeats (KIV-2 repeat polymorphism) that is responsible for the apo(a) isoform variation [1–3, 5•]. Plasma Lp(a) levels vary widely among individuals, are primarily genetically determined by variation in the LPA gene coding for apo(a), and are inversely correlated with the apo(a) size [1–3, 5•].

Mendelian randomization studies, large epidemiological databases, and genome-wide association studies linking genetically determined Lp(a) levels to atherosclerotic cardiovascular disease (ASCVD) events have established that Lp(a) is a causal risk factor for ASCVD, independent of other conventional risk factors [5•, 6–8].

In the present review article, the pathophysiological role and clinical significance of Lp(a) are briefly presented. Subsequently, the effects of current hypolipidemic therapies on Lp(a) levels are discussed, followed by the presentation of the pharmacodynamic effectiveness in reducing Lp(a) levels as well the clinical efficacy and safety of drugs that specifically target Lp(a) and are currently under investigation. The technologies on which the development of these new drugs is based are also briefly presented.

## Methods

The databases MEDLINE, EMBASE, and CENTRAL were searched up to June 30, 2023, using the following keywords: antisense oligonucleotides (ASOs), argonaute proteins, atherosclerosis, bempedoic acid, calcific aortic valve stenosis, cardiovascular risk, CETP inhibitors, CRISPR/Cas9, genome editing, ezetimibe, fibrates, hypolipidemic drugs, lipoprotein apheresis, lipoprotein(a), niacin, oxidized phospholipids, RNA interference, small interfering RNAs, proprotein convertase subtilisin/kexin type 9 inhibitors, statins. Research and review articles, case reports, and clinical trials were assessed, whereas the references of these articles were scrutinized for other relevant articles.

### Lp(a) Pathophysiological Role and Clinical Significance

Lp(a) is found in human atherosclerotic plaques and a higher content of Lp(a) correlates with the severity of ASCVD clinical presentation [9, 10]. Lp(a) mediates atherogenicity via multiple mechanisms, in which a variety of Lp(a)-associated factors are implicated [9–11]. These include monocyte chemoattractant protein-1 (MCP-1), which mediates the Lp(a)-induced enhancement of monocytes trafficking and recruitment to the lesion site [12], β2-integrin Mac-1 which interacts with apo(a) and contributes to the Lp(a)-mediated promotion of monocytes adhesion and transendothelial migration [13], autotaxin (ATX), a secreted enzyme that exhibits a lysophospholipase D activity and is preferentially transported by Lp(a) [14, 15]. ATX catalyzes the hydrolysis of lyso-PC into lysophosphatidic acid (Lyso-PA), which stimulates complex intracellular signaling pathways through its G coupled–receptor [14–16]. Lp(a) is also the main transporter of oxidized phospholipids (OxPL), which are generated by the oxidation of polyunsaturated fatty acid residues esterified at the *sn*-2 position of phospholipids and play key roles in the Lp(a) functionality [4, 17, 18].

In addition to its important role in ASCVD, Lp(a) may also play a causal role in calcific aortic valve stenosis (CAVS) [4, 19, 20]. In this regard, a genetic variant (rs10455872) in the *LPA* gene locus, which is an important determinant of plasma Lp(a) levels, is causally related to CAVS [21]. Furthermore, the ASTRONOMER (Aortic Stenosis Progression Observation: Measuring Effects of Rosuvastatin) trial analysis demonstrated that elevated Lp(a) and OxPL/apoB levels are independently associated with an increased risk of aortic stenosis progression rate [22]. Accordingly, other studies demonstrated that elevated Lp(a) and OxPL/ApoB are independently associated with increased aortic valve calcification and CAVS progression as well as with an increased incidence of aortic valve replacement and death [23–26].

Overall, Lp(a), and its associated OxPL, ATX, and Lyso-PA play important roles in the pathogenesis of ASCVD and CAVS and provide a rationale for therapeutic approaches aiming to reduce the risk of these chronic disorders.

### Effect of Current Hypolipidemic Drugs on Plasma Lp(a) Levels

The currently used hypolipidemic drugs only modestly affect the plasma Lp(a) as well as OxPL/ApoB levels (Table 1) [27]. In this regard, several studies [28, 29] and a meta-analysis [30] have demonstrated that statins increase Lp(a) and OxPL/ApoB levels compared with placebo (Table 1). Both baseline and on-statin Lp(a) plasma levels were associated with an almost linear increase in CVD events, particularly in patients with Lp(a) levels >50 mg/dL [27, 31]. As concern fibrates, a meta-analysis showed that they modestly reduce Lp(a) levels [32] Combination therapy with a statin and a fibrate reduce plasma Lp(a) levels to a similar extent to that observed with fibrate monotherapy, this reduction being greater to that induced with a statin monotherapy [27, 33]. Niacin, mipomersen, lomitapide, cholesteryl ester transfer protein (CETP) inhibitors, and proprotein convertase subtilisin kexin 9 (PCSK9) inhibitors (alirocumab, evolocumab, and inclisiran), also modestly reduce plasma Lp(a) levels [27, 33]. Ezetimibe monotherapy exhibits a small (approximately 7%), but significant decrease in Lp(a) levels [34]. Overall, all the above therapeutic interventions have a modest, effect in reducing Lp(a) plasma levels (Table 1), suggesting modest regulatory influences on LPA gene expression, Lp(a) assembly, and/or clearance).

**Table 1:** The effect of current hypolipidemic therapies on plasma Lp(a) levels

Lp(a)-lowering therapy	Plasma Lp(a) levels	Plasma OxPL/apoB levels
Statins	6.5–26.0% increase (mean 10.6%)	8–48% increase (mean 23.8%)
Ezetimibe	Approximately 7% reduction	Unknown effect
Niacin	20–30% reduction	Approximately 17% reduction
Fibrates	Up to 25% reduction	Unknown effect
CETP inhibitors	10–40% reduction	Unknown effect
PCSK9 inhibitors	10–30% reduction	Unknown effect
Lp(a) apheresis	70–75% acute reduction, 25–40% mean reduction	50–75% reduction
Mipomersen	21–39% reduction	10–30% reduction
Lomitapide	15% reduction	Unknown effect

Abbreviations: *PCSK9* inhibitors, proprotein convertase subtilisin kexin 9 inhibitors; *CETP inhibitors*, cholesteryl ester transfer protein inhibitors

Lipoprotein apheresis (LA) induces a significant acute reduction of plasma Lp(a) levels by 70–75% [35]. However, a rapid rebound of Lp(a) levels to baseline is observed between apheretic sessions [36]. Thus, weekly or biweekly, an interval mean Lp(a) reduction of 25–40% is observed, depending on LA course and on baseline Lp(a) levels [37]. Long-term studies in patients with high Lp(a) levels undergoing LA suggest that this therapy may reduce the 5-year ASCVD risk by up to 86% [38, 39].

Do the modest reductions of Lp(a) levels induced by the current hypolipidemic drugs, have a clinical impact regarding cardiovascular risk? In the FOURIER trial, evolocumab decreased Lp(a) levels by 12 mg/dL and it corresponded to a 15% reduction, in the relative ASCVD risk [40]. Furthermore, in the ODYSSEY OUTCOMES trial, the Lp(a) reduction by 25% with alirocumab was associated with an up to 38% reduction of major adverse cardiovascular events (MACEs) (non-fatal myocardial infarction, peripheral arterial disease, and venous thromboembolism), independently on alirocumab-induced decrease of other atherogenic lipoproteins [41].

In contrast to the results of the above studies on PCSK9 inhibitors, which showed that modest decreases of plasma Lp(a) levels are associated with reductions of ASCVD risk, a Mendelian randomization analysis demonstrated that in order to achieve an ASCVD risk reduction by 20%, as that observed with LDL-cholesterol lowering by 38.67 mg/dL, a 101.5 mg/dL Lp(a) reduction is required [42]. Subsequent analyses using a similar methodology demonstrated that the Lp(a) reduction in order to achieve a 20% ASCVD risk reduction, should be 65.7 mg/dL [43] or 50 mg/dL [44]. It is noted that the above studies evaluated populations in the “primary prevention” state, and therefore their relevance to outcome trials of secondary prevention is not strong enough. However, as has been already mentioned above, all secondary prevention trials including the ODYSSEY OUTCOMES trial, support the suggestion that the existing hypolipidemic therapies targeting the traditional lipid risk factors (LDL-cholesterol and triglycerides) are not adequate to reduce Lp(a) levels to such an extent that could lead to a substantial reduction of the residual ASCVD risk attributed to elevated Lp(a) plasma concentrations. Consequently, the scientific community is not ready today to efficiently treat high Lp(a) levels and there is an unmet need for new drugs that will specifically target Lp(a) and potentially reduce its levels and the ASCVD risk. Such agents are currently under investigation and are based on RNA and gene editing technologies. These technologies as well as the results of recent pharmacodynamic and clinical studies are presented below.

### RNA Technologies for Drug Development

#### The Antisence Oligonucleotide Technology

**Fig. 2:**
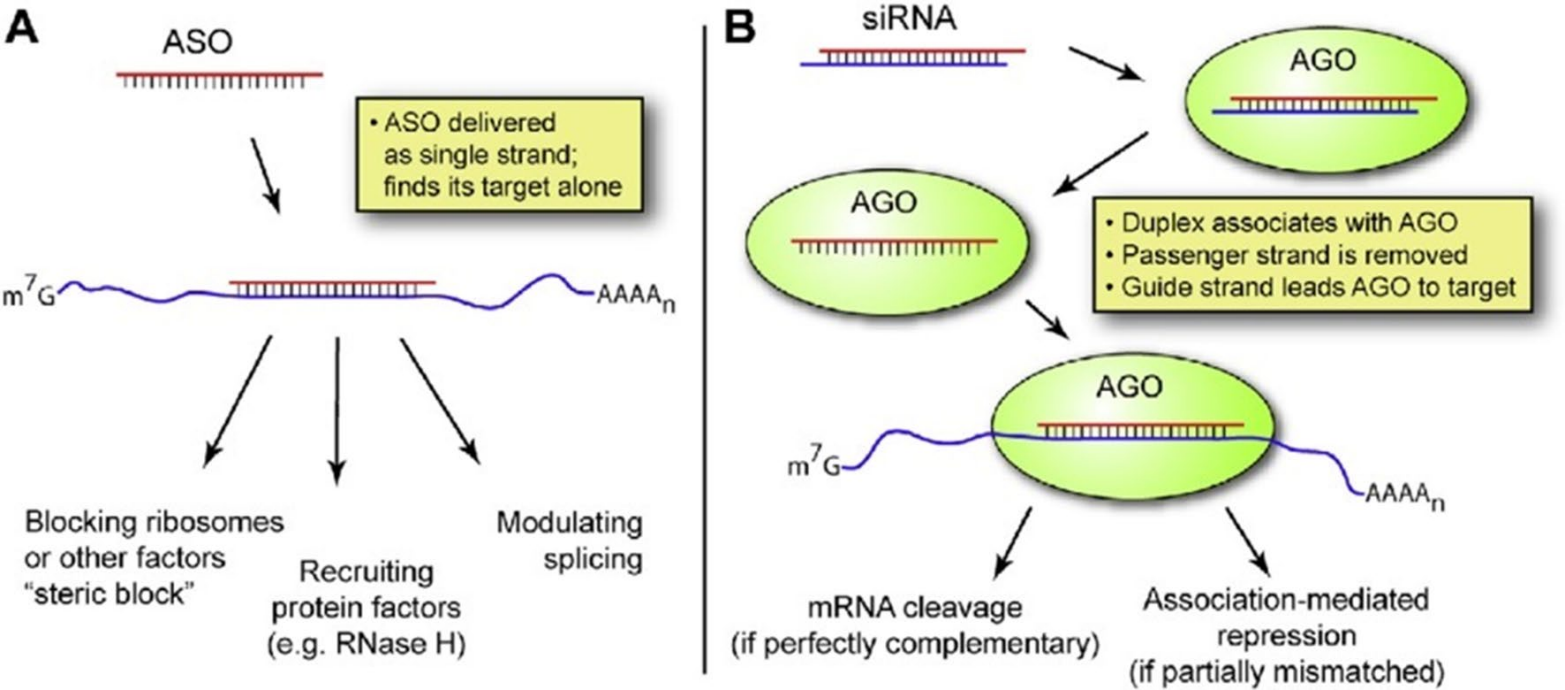
RNA technologies for drug development. **A** The antisence oligonucleotide (ASO) technology. ASO modulates intracellularly the stability, processing, or activity of the bound mRNA through various mechanisms. Τhe formed ASO-mRNA complex triggers ribonuclease H (RNase H) activity leading to mRNA degradation. ASO-mRNA complex also induces translational arrest by steric hindrance of ribosomal activity. Furthermore, it interferes with mRNA maturation by inhibiting splicing or it destabilizes pre-mRNA in the nucleus**. B** The RNA interference mechanism. siRNAs are delivered as duplexes and are taken up by Argonaute (AGO) proteins, part of the RNA-induced silencing complex (RISK). siRNA is then separated into two single-stranded RNAs, the passenger, and the guide strand. The guide strand binds the target mRNA and induces its cleavage by AGO proteins. (Reprinted from *Arch Med Res*. 2018 Nov;49(8):538-547. doi: 10.1016/j.arcmed.2019.01.001, with permission from Elsevier) [49]

Antisense oligonucleotides (ASOs) are short-strand (16- to 20-nucleic-acid-long) DNA fragments that bind to their complementary mRNA target via Watson-Crick A-T, C-G base pairing [45–48]. Subsequently, ASO modulates intracellularly the stability, processing, or activity of the bound mRNA through various mechanisms (Fig. 2A). Among them, the formed ASO-mRNA complex triggers ribonuclease H1 (RNase H1) activity leading to mRNA degradation [50]. This results in lowering mRNA levels therefore it leads to a significant inhibition of target protein synthesis. ASO-mRNA complex also induces translational arrest by steric hindrance of ribosomal activity, it interferes with mRNA maturation by inhibiting splicing or it destabilizes pre-mRNA in the nucleus (Fig. 2A) [45–48].

ASOs technology is used to specifically inhibit protein synthesis and is considered an efficacious, therapeutic strategy [46, 51]. To improve the ASO resistance to nucleases, to increase their half-life and affinity for the target mRNA and to reduce toxicity they undergo chemical modifications. In this regard, phosphorothioate modification to the phosphodiester backbone of ASOs (PS-ASOs) increases their resistance to nucleases but lowers their specificity and rate of cellular uptake. 2’-O-Methyl (2’-OMe), and 2’-O-methoxyethyl (2’-OMOE), modifications of PS-ASOs increase their binding affinity for target mRNA. Other modifications include the formulation of the peptide nucleic acid (PNA), the locked nucleic acid (LNA), and the phosphoroamidate morpholino oligomer (PMO), performed by chemical modifications of the furanose ring of ASOs nucleotides [52, 53]. Furthermore, to enhance the specific binding of ASOs to the hepatocyte asialoglycoprotein receptor (ASGPR) and therefore their specific delivery to hepatocytes, conjugates of ASOs, phosphodiester-linked with triantennary N-acetyl-galactosamine (GalNAc), a highly efficient ligand for the ASGPR, were developed. Upon binding to the receptor, the ASO-ASGPR complex is transferred into hepatocytes by clathrin-mediated endocytosis. ASO is then released from ASGPR, and ASGPR recycles to the hepatocyte surface. These ASO-GalNAc conjugates exhibit a 10-fold greater potency than ASOs without the conjugate [52–54]. The ASOs technology is considered a very promising new therapeutic approach to efficiently reduce plasma Lp(a) levels (described below).

#### The RNA Interference Mechanism and the Argonaute Proteins

The Argonaute (AGO) proteins, play an important role in RNA silencing processes. The AGO gene family encodes for six characteristic domains: N- terminal, Linker-1, PAZ, Linker-2, Mid, and a C-terminal PIWI domain [55].

AGO proteins bind different classes of small non-coding RNAs, including microRNAs (miRNAs), small interference RNAs (siRNAs), and Piwi-interacting RNAs (piRNAs).

The PAZ domain of AGO is an RNA binding module that recognizes single-stranded 3′ ends of siRNA, miRNA, and piRNA, in a sequence-independent manner. The PIWI domain is essential for the target cleavage. It mediates protein-protein interaction and binds to Dicer at one of the RNase III domains [56].

In humans, there are eight AGO family members. However, endonuclease activity and thus RNAi-dependent gene silencing is exclusively mediated by AGO2.

AGO proteins are guided by small RNAs to their specific targets through sequence complementarity (base pairing), which then leads to specific mRNA cleavage and therefore to translation inhibition [57]. AGO proteins are essential components of the RNA-induced silencing complex (RISC), and represent the active part of RISC, cleaving the target mRNA strand complementary to siRNA bound to these proteins [58].

RISC is responsible for gene silencing, a mechanism known as RNA interference (RNAi).

RNAi is a biological process through which RNAs inhibit gene expression, either via the destruction of specific mRNA molecules or by suppressing the protein translation [59]. The RNAi is an important mechanism that cells use to defend against parasitic nucleotide sequences. The RNAi pathway is initiated by the enzyme Dicer which cleaves long double-stranded RNAs (dsRNAs) into short double-stranded fragments of about 20 nucleotide siRNAs. The dsRNA is then separated into two single-stranded RNAs, the passenger and the guide strand. The passenger strand is degraded, while the guide strand is incorporated into the RISC, where it binds the target mRNA and induces cleavage by AGO proteins, existing within the RISC, thus leading to reduced protein synthesis [60]. The RNAi pathway is currently used for pharmaceutical purposes, via the design synthesis and administration *in vivo* of siRNAs to specifically reduce the synthesis of the target protein, this reduction lasting for several weeks after administration of a single siRNA dose. Synthetic siRNAs have polyanionic nature and exhibit high molecular weight, therefore they require delivery platforms to cross the membranes and access the cytoplasm of target cells. Efficient delivery platforms for systemic siRNA administration are lipid nanoparticles (LNPs), which contain ionizable amino lipids that interact with polyanionic nucleic acids and form nanoparticles encapsulating the siRNA molecule [48, 61]. Following LNPs administration, these particles are endocytosed by target cells and subsequently, their siRNA content escapes the endosomes and accesses the cell cytoplasm [48, 61].

### Genome Editing Using CRISPR/Cas9

The clustered regularly interspaced short palindromic repeat (CRISPR) / CRISPR-associated protein (Cas) system has facilitated the last years development of new therapies based on genome editing [62]. Cas9 is an RNA-guided DNA endonuclease associated with the CRISPR adaptive immunity system in *Streptococcus pyogenes* and various other bacteria [63]. Cas9 has gained traction in recent years because it can cleave nearly any sequence complementary to the guide RNA [64]. To achieve site-specific DNA recognition and cleavage, Cas9 must be complexed with both a CRISP RNA (crRNA) and a separate trans-activating crRNA (trRNA) that is partially complementary to the crRNA [64]. In light of the observed high efficiencies of CRISPR-Cas9 in mammalian cells *in vitro* [65, 66] genome editing offers a highly attractive alternative approach to efficiently reduce the hereditary high levels of various risk factors including Lp(a), offering a lifelong therapeutic effect with only a single administration [65, 66].

### Emerging Therapies That Potently Reduce Lp(a) Levels

As it is described above, currently available approaches, including hypolipidemic therapies, only modestly reduce plasma Lp(a) levels [27]. This has led to intensive research efforts to develop agents that target the mRNA transcript of LPA, the gene encoding apo(a) [15], which could efficiently inhibit Lp(a) synthesis leading to potent reduction of Lp(a) levels. These new therapeutic approaches use investigational ASOs or siRNAs agents that have been tested for their pharmacodynamic and safety profile and are currently under investigation in clinical trials to determine their efficacy, tolerability in Lp(a)-lowering and their potential effectiveness in reducing residual CVD risk attributed to high plasma Lp(a) levels.

#### ASOs Targeting the apo(a) mRNA

ASOs designed to induce the degradation of the mRNA coding for apo(a), inhibit apo(a) synthesis in the liver, and specifically and potently reduce Lp(a) secretion and consequently its plasma levels. These ASOs are injected subcutaneously, bind to plasma proteins, and enter the liver cells. They bind intracellularly to the apo(a) mRNA forming a double-stranded complex, primarily in the nucleus but also in the cytoplasm. The sense strand is degraded by the RNase H1 thus preventing the synthesis of apo(a) and therefore the Lp(a) assembly, leading to the significant reduction of plasma Lp(a) levels (Fig. 2A). It should be noted that the inhibition of Lp(a) formation, does not affect the synthesis and secretion of very low-density lipoprotein (VLDL) and LDL and therefore their plasma levels [4, 7].

Investigational ASOs to apo(a) mRNA are being tested in clinical trials to determine their Lp(a)-lowering efficacy, safety, and tolerability, and their clinical effectiveness in reducing CVD risk (Table 2).

**Table 2:** Emerging therapies that potently reduce plasma Lp(a) levels

Specific Lp(a)-lowering Drugs	Mean Reduction of Plasma Lp(a) levels	Mean Reduction of Plasma OxPL/apoB levels
ASOs		
ISIS-APO(a)Rx (IONIS-APO(a)Rx	No significant with single doses 39.6–77.8% with multiple doses	35.5–78.1% with multiple doses
Pelacarsen (IONIS-APO(a)-LRx, TQJ230)	26–85% with single doses 66–92.4% with multiple doses	77–85% with multiple doses
siRNAs		
Olpasiran (AMG-890)	66.9–97.5% with multiple doses	Unknown effect
SLN360	46–98% with multiple doses	Unknown effect
CRISPR/Cas9 Lp(a) genome editing	Nearly complete elimination of apo(a) from the circulation within a week*	Unknown effect

*Study performed in LPA transgenic mouse model expressing apo(a)

Abbreviations: *ASOs*, antisense oligonucleotides; *CRISPR/Cas*, clustered regularly interspaced short palindromic repeat (CRISPR) / CRISPR-associated protein (Cas); *siRNA*, small interference RNA

IONIS-APO(a)Rx phase I study investigated the efficacy of a specific ASO to apo(a), which contains 20 nucleotides and designated as IONIS-APO(a)Rx, in 47 healthy volunteers aged 18–65 years with Lp(a) concentrations >100 mg/dL. Participants were randomized to receive either one single dose of IONIS-APO(a)Rx or placebo administered subcutaneously at varying concentrations (50–400 mg) or 6 consecutive doses at varying concentrations or placebo. The multiple-dose treatment produced a substantial dose-dependent reduction in Lp(a) levels from baseline to the end of the 5th week (39.6–77.8%). Significant reductions in the levels of OxPLs carried by Lp(a) were also noted at week 5 (a decrease of up to 78%) [67].

In a phase II trial of IONIS-APO(a)Rx, 2 groups of patients with elevated Lp(a), cohort A with Lp(a) levels 125 to 437 nmol/l, and cohort B with Lp(a) levels 438 nmol/l, were participated [68, 69]. Patients received subcutaneously for 1 month, 100 mg, 200 mg, and 300 mg weekly of IONIS-APO(a)Rx, for a total duration of 3 months. In both cohorts, similar percent reductions in Lp(a) were observed, but the absolute reduction in Lp(a) was higher in cohort B (183 vs. 305 nmol/L or 73 vs. 122 mg/dL) [69]. Significant reductions in OxPL carried by Lp(a) expressed as OxPL-apoB or OxPL-apo(a) as well as LDL-C and apoB-100 were also observed in both cohorts [68]. Finally, a reduced monocyte inflammatory activation that returned close to baseline levels after stopping treatment was also observed [69].

Based on these promising results, the IONIS-APO(a)Rx ASO was modified by the conjugation of a GalNac3 molecule. This enables its specific binding to the liver ASGPR and selective uptake, at high rates, by hepatocytes [68]. This provides increased drug potency, less-frequent dosing, and minimum toxicity [68]. Furthermore, in this modified ASO, 6 of the 19 phosphorothioate linkages were replaced with phosphodiester linkages at positions 2, 3, 4, 5, 16, and 17. The ASO-GalNac3 was initially designated as IONIS-APO(a)-LRx, AKCEA-APO(a)-LRX, or TQJ230, and subsequently as Pelacarsen [68].

Its efficacy in reducing Lp(a) levels, was studied in a phase 1/2a trial. Pelacarsen was injected subcutaneously in 58 healthy volunteers with Lp(a) levels ≥ 75nmol/L. Participants were randomly assigned to receive single doses of 10, 20, 40, 80, and 120 mg or multiple doses of 10, 20, or 40 mg of pelacarsen. Significant dose-dependent reductions in mean Lp(a) concentrations ranging from 26.2% to 85.3% were observed in all single-dose pelacarsen groups at 1 month [68]. In the multidose groups, pelacarsen induced mean Lp(a) reductions of 66–92% in a dose-dependent manner. Significant reductions were also observed in OxPL-apoB, OxPL-apo(a), LDL-C, and apoB-100 levels. The above changes were independent of the Lp(a) isoform size [68].

In a phase IIb randomized, double-blind, placebo-controlled, dose-ranging trial 286 CVD patients with Lp(a) >60 mg/dL (>150 nmol/L) participated. The majority of patients were <65 years old, and approximately one-half had premature coronary artery disease and prior to myocardial infarction. Patients were receiving hypolipidemic therapy and had well-controlled LDL-C levels. The study population was randomized to receive subcutaneously varying doses of Pelacarsen, 20, 40, or 60 mg every 4 weeks, 20 mg every 2 weeks, and 20 mg every week, or a placebo for 6 to 12 months [70]. Pelacarsen reduced Lp(a) levels by 35–80% in a dose-dependent manner (compared with 6% in the placebo group). The maximum reduction in Lp(a) levels was observed at 14 weeks of treatment and was then sustained throughout the duration of treatment. This study showed that approximately 98% of patients receiving the 80 mg monthly dose regimen achieved Lp(a) levels <50 mg/dL, the established threshold for Lp(a)-driven CVD [31, 71]. Significant dose-dependent reductions in apoB, OxPL-apoB, OxPL-apo(a) and LDL-C, from baseline were also observed [70].

Finally in a more recent study, the effect of pelacarsen on directly measured Lp(a)-cholesterol (Lp(a)-C) and LDL-C, corrected for its Lp(a)-C content, in patients with a history of CVD and elevated Lp(a) randomized to 5 groups of cumulative monthly doses of 20–80 mg pelacarsen vs placebo, was studied. The baseline median Lp(a)-C values in the groups ranged from 11.9 to 15.6 mg/dL, the LDL-C ranged from 68.5 to 89.5 mg/dL and the LDL-Ccorr ranged from 55 to 74 mg/dL. Pelacarsen significantly reduced in dose-dependent manner Lp(a)-C, compared with placebo (29 to 67% vs 2%) whereas it had a neutral to mild lowering effect on LDL-Ccorr. Furthermore, these results suggest that in patients with elevated Lp(a), LDL-Ccorr provides a more accurate reflection of changes in LDL-C than other methods used to determine LDL-C [72].

It remains to be established whether Pelacarsen will reduce CVD events related to high Lp(a). This is currently studied in a phase III randomized double-blind, placebo-controlled, multicenter trial clinical trial (HORIZON trial; NCT04023552). This trial investigates the impact of Lp(a) lowering with 80 mg Pelacarsen injected monthly subcutaneously vs placebo on major cardiovascular events in 7680 patients with Lp(a) ≥70 mg/dL (≥175 nmol/L) and a history of prior myocardial infarction, stroke, or symptomatic peripheral artery disease. The estimated study completion date is May 30, 2025.

#### Lp(a) Lowering with siRNAs

1. **Olpasiran**. Similar to ASOs, GalNAc conjugation has also been used in developing siRNA agents to silence apo(a) mRNA in hepatocytes (Fig. 2B). Olpasiran (formerly designated as AMG-890) is a GalNAc-conjugated siRNA drug (Fig. 2B). Following a series of preclinical experiments designed to characterize the pharmacokinetic and pharmacodynamic effects of olpasiran in transgenic mice and cynomolgus monkeys [73], a subsequent phase 1, first-in-human clinical study was designed to evaluate the safety and tolerability, as well as the pharmacokinetics and pharmacodynamics, of single subcutaneous doses (3 to 75 mg) of olpasiran versus placebo in individuals with elevated Lp(a) [73]. The study included 7 cohorts of subjects with Lp(a) levels ≥70 nmol/L to ≤199 nmol/L or ≥200 nmol/L. In the group with Lp(a) levels of 70 to 199 nmol/l, a reduction from baseline levels of Lp(a) by 71 to 96% at day 43, and by 80 to 94% at day 113 (cohorts 2 to 5) was observed with olpasiran. In the Lp(a) of 200 nmol/l group, olpasiran reduced mean Lp(a) levels from baseline by 75 to 89% at day 43 and by 61 to 80% at day 113. No safety concerns were reported [73]. In an effort to determine the optimal dosing and design for a cardiovascular outcomes trial with olpasiran, the Olpasiran trials of Cardiovascular Events And lipoproteiN(a) reduction-DOSE finding (OCEAN(a)-DOSE trial) was designed to assess the Lp(a)-lowering efficacy and safety of olpasiran [74]. The OCEAN(a)-DOSE study was a phase 2 multicenter, randomized, double-blind, placebo-controlled dose-ranging study in 281 patients with established ASCVD and Lp(a) > 150 nmol/L. Patients were randomly assigned to receive one of four subcutaneous doses of olpasiran (10 mg every 12 weeks, 75 mg every 12 weeks, 225 mg every 12 weeks, or 225 mg every 24 weeks) or a matching placebo. The primary end point was the percent change in Lp(a) levels from baseline to week 36. The drug safety was also assessed [74]. The OCEAN(a)-DOSE trial included 281 patients, exhibiting 260.3 nmol/L median Lp(a) concentration at baseline [75]. Among patients, 88% were taking statin therapy, 52% were taking ezetimibe, and 23% were taking a PCSK9 inhibitor, consequently the LDL-C levels in all patients were well controlled (median concentration 67.5 mg/dL) [75]. Olpasiran treatment for 36 weeks, significantly reduced the Lp(a) levels in a dose-dependent manner (placebo-adjusted mean percent reductions of 70.5% with the 10mg dose, 97.4% with the 75mg dose, 101.1% with the 225mg dose administered every 12 weeks, and 100.5% reduction with the 225mg dose administered every 24 weeks. In the placebo group, Lp(a) increased by a mean of 3.6%. The overall incidence of adverse events was similar across all groups, whereas the most common olpasiran-related adverse events were injection site reactions, primarily pain [75]. These very promising results will be the base for designing new trials of longer duration involving a larger number of participants in order to definitively establish the clinical efficacy and safety of olpasiran in ASCVD patients. In this regard, the OCEAN(a) -Outcomes Trial is an ongoing Phase 3 double-blind, randomized, placebo-controlled, multicenter study, which will investigate the impact of olpasiran (sc injection once every 12 weeks) on major cardiovascular events in participants with ASCVD and Lp(a) ≥ 200 nmol/L during screening. The estimated study completion date is December 2026.
2. **SLN360**. This drug is a 19-mer GalNAc-conjugated siRNA. The phase 1 APOLLO trial examined the tolerability and safety of SLN360 following a single dose in 32 adults with Lp(a) plasma concentrations of 150 nmol/L or greater at screening and no known clinically overt CVD. The authors also assessed associated changes in plasma concentrations of Lp(a) at different doses to a maximum follow-up of 150 days. Participants were randomized to receive placebo (*n* = 8) or single doses of SLN360 at 30 mg (*n* = 6), 100 mg (*n* = 6), 300 mg (*n* = 6), or 600 mg (*n* = 6), administered subcutaneously [76]. Results showed that SLN360 was well tolerated and induced a dose-dependent lowering of plasma Lp(a) concentrations. Lp(a) levels over 150 days were reduced by 10% in the placebo group and by 46%, 86%, 96%, and 98% in the 30-mg, 100-mg, 300-mg, and 600-mg SLN360 groups, respectively. These support further studies to determine the safety and efficacy of SLN360 [76].

#### CRISPR/Cas9 Lp(a) Genome Editing

Somatic genome editing has the potential to be a one-time therapy for individuals with extremely high Lp(a) levels. To address this hypothesis an LPA transgenic mouse model expressing apo(a) of physiologically relevant size was generated [77]. Adeno-associated virus (AAV) vector delivery of CRISPR/Cas9 was used to disrupt the LPA transgene in the liver [77]. Authors demonstrated that AAV-CRISPR/Cas9 nearly completely eliminated apo(a) from circulation within a week. This proof-of-concept study establishes the feasibility of using CRISPR-Cas9 to disrupt LPA *in vivo* [77]. This is the first study performed in an LPA transgenic mouse model expressing apo(a) demonstrating a successful *in vivo* editing of LPA and suggests that one-time treatment with a gene-editing nuclease could provide permanent removal of Lp(a) in patients at high residual CVD risk attributed to Lp(a). The results of this study are a valuable proof of concept for the development of a new class of Lp(a) therapeutics. However, this remains to be evaluated in humans in future studies.

## Conclusion and Future Perspectives

To date, the hypolipidemic drugs used in daily clinical practice only modestly affect Lp(a) levels and do not have a clinical benefit in ASCVD risk to the extent observed with the reduction of LDL-cholesterol and Triglyceride levels. Neither these therapies reduce the risk of CAVS. Thus, we are not ready today to specifically and potently reduce Lp(a) levels. Furthermore, it is not known whether potent and long-term decreases in Lp(a) levels will be translated into important reductions of ASCVD or CAVS risk. Thus, it is not possible today to draw safe conclusions on the clinical significance of specifically reducing Lp(a) levels. The results of the recent studies on the new specific anti-Lp(a) drugs are very promising. However, their clinical benefit needs to be evaluated in phase III clinical trials. In conclusion, today we are not ready to efficiently treat high Lp(a) levels and we should wait for the results of ongoing clinical trials. If the novel Lp(a)-lowering agents are proven to be clinically effective and safe, they hold the potential to have an important beneficial impact on the health of patients, their families, and the wider population exhibiting genetically determined high Lp(a) levels.
